# COPD in never-smokers: evidence, challenges, and knowledge gaps

**DOI:** 10.48101/ujms.v131.14704

**Published:** 2026-10-27

**Authors:** Pernilla Sönnerfors, Magnus Sköld

**Affiliations:** a Department of Medicine Solna, Division of Immunology and Respiratory Medicine, Center for Molecular Medicine, Karolinska Institutet, Stockholm, Sweden; b Medical Unit Occupational Therapy and Physiotherapy, Women’s Health and Allied Health Professionals Theme, Karolinska University Hospital, Stockholm, Sweden; c Department of Respiratory Medicine and Allergy, Karolinska University Hospital, Stockholm, Sweden

**Keywords:** COPD never-smokers, Clinical phenotypes, Clinical characteristics, Airway obstruction, Early life events, Lung function trajectories, Risk factors

## Abstract

**Objectives:**

This study aimed to summarise current evidence on chronic obstructive pulmonary disease (COPD) in never-smokers, including definitions, epidemiology, risk factors, clinical characteristics, and emerging conceptual frameworks, and identify key knowledge gaps.

**Results:**

Although tobacco smoking remains the predominant global risk factor for COPD, a substantial proportion of individuals with COPD are lifelong never-smokers. International data indicate that 22–51% of people with spirometry-defined COPD have never smoked, with a global COPD prevalence of 4–16% in this group. Risk factors span through the life course and include impaired lung development, early-life disadvantages, recurrent respiratory infections, asthma, indoor and outdoor air pollution, occupational exposures, environmental tobacco smoke, socioeconomic disadvantages, ageing, and genetic susceptibility. Research remains limited as most studies have historically focused on smoking-related COPD.

Never-smokers with COPD show several distinct clinical features compared with those with tobacco-related disease. They tend to have an earlier age of onset, less severe airflow obstruction, and a higher prevalence of small airways disease, particularly in individuals with a history of asthma. Symptoms such as cough, sputum production, and exertional breathlessness are reported less frequently. Physiologically, never-smokers commonly demonstrate normal diffusing capacity, less emphysema, and a slower decline in lung function. Respiratory comorbidities such as asthma and allergic disease occur more often, suggesting differing inflammatory pathways. Fewer never-smokers meet GOLD stage III–IV criteria. Despite increasing recognition, COPD in never-smokers remains insufficiently characterised. Approximately one third of global COPD cases occur in never-smokers, yet they are routinely excluded from clinical trials, limiting understanding of mechanisms, natural history, and treatment response.

**Conclusions:**

Never-smoker COPD represents a distinct and understudied phenotype with unique risk factors, clinical features, and comorbidity patterns. Improved diagnostic strategies, phenotyping, and dedicated clinical trials are needed to guide personalised management for this population.

## Introduction

Chronic obstructive pulmonary disease (COPD) is associated with a wide range of risk factors, among which tobacco smoking is the most common (1, 2). However, accumulating evidence indicates that a considerable proportion of individuals with COPD are lifelong non-smokers. This group remains insufficiently characterised and there is a need for more knowledge on risk factors, epidemiology, clinical behaviour, and outcome (3–5).

According to the World Health Organization (WHO), globally, the prevalence of tobacco uses among individuals aged 15 years and older in 2025 was estimated at 19.8%, with a marked sex difference: 32.9% among males and 6.7% among females. In 2022, the proportion of males using any form of tobacco was more than four times that of females, underscoring the persistent gender disparity in tobacco use worldwide (6). Sweden has one of the lowest smoking prevalences globally. In 2024, only 5.4% of adults (aged 16–84) reported daily smoking, placing the country close to the WHO’s threshold for a ‘smoke-free’ society, which is under 5%. Although daily smoking in Sweden has declined, the overall nicotine use is as high as 30%, which is driven by a significant use of snus and e-cigarettes (7). In high-income countries, tobacco smoking remains the predominant contributor, responsible for about 70% of COPD cases. In low- and middle-income countries, by contrast, tobacco smoking is estimated to account for only 30% to 40% of the total COPD burden (8). As these countries collectively represent more than 85% of the global COPD burden, non-smoking-related risk factors now contribute to more than half of the worldwide burden of COPD (8).

This review will summaries the current knowledge on COPD in never-smokers and address knowledge gaps for future research.

## COPD in never-smokers: definitions

The WHO defines never-smokers as individuals with no history of regular daily smoking and who have smoked less than 100 cigarettes, or the equivalent amount of tobacco, over their lifetime (9). Objective assessments of smoking history often relies on biomarkers such as serum cotinine levels (10) and/or exhaled carbon monoxide levels, however these measures only assess recent exposure or current use of cigarettes and other nicotine products (11). In contrast, self-reported smoking status often leads to an under-estimation of actual smoking prevalence and smoking behaviours (12). Therefore the 100-cigarette lifetime threshold could produce underestimates of the relative harms from tobacco smoking and this should be taken into account in reports on ‘never-smokers’ (13).

A diagnosis of COPD should be considered in patients with dyspnoea, chronic cough, sputum production, recurrent lower respiratory tract infections, or relevant exposure to risk factors. While symptoms are common, they are not mandatory to establish a COPD diagnosis. However, a post-bronchodilator spirometry revealing chronic airflow limitation (CAL) is required. In the Global Initiative for Chronic Obstructive Lung Disease (GOLD) document, this is defined as a post-bronchodilator FEV_1_/FVC ratio < 0.70. (14). Since CAL may be underestimated in younger age and overestimated in the elderly population, lower limit of normal (LLN) is an alternative to assess whether CAL exists (15).

Most studies published on never-smokers with COPD have used the post-bronchodilator FEV_1_/FVC < 0.7 definition for COPD (3–5, 16–19) or the FEV_1_/FVC < 0.7 together with ratio < LLN, and /or FEV_1_ predicted values (20–23), but some used pre-bronchodilator FEV_1_/FVC < 0.7 and < LNN (24, 25). The definition used in studies for participants being never-smokers is often not described more thoroughly than participants being ‘non-smokers’ or ‘never-smokers’ or having ‘no prior smoking history at the time of the study enrollment’ (19) or when described, the WHO definition is used; < 100 cigarettes or equivalent amount of tobacco (21).

## Epidemiology

Estimating the prevalence of COPD is challenging because different diagnostic criteria can yield various results. Age and demographic factors also influence test interpretation and spirometry remains underused globally. Understanding these limitations help us to explain why published COPD prevalence estimates vary widely between studies and regions (26). The global prevalence of COPD in adults is estimated to 10% (2). Epidemiological data, most using only spirometry-based COPD definition, have shown that a broad range (22–51%) of people affected by COPD have never smoked (8). Furthermore, a recent review reported that the global prevalence of COPD among never-smokers was 4–16% (8, 27) worldwide and in Sweden 3,0–7.7 %. Never-smokers constituted 7–51% of all people diagnosed with COPD and was equally distributed among men and women (8).

The Lancet Commission on COPD recently proposed a classification of the disease into five categories based on its predominant risk factors: genetic causes, COPD due to abnormal lung development, infection-related COPD, smoking- or vaping-associated COPD, and COPD attributable to environmental exposures (28). Based on this classifications, a revised COPD criteria was presented in the GOLD document (14). In these guidelines, a smoking history as a major cause is toned down, to acknowledge the heterogeneity of the disease. COPD, as an umbrella term, is suggested to be categorised in six etiotypes (14). Consequently, the term COPD includes individuals both with and without a history of tobacco smoking, and current guidelines recognise several never-smoking related forms of COPD. These non-tobacco-related etiotypes include the categories above, but also asthma-associated, and COPD of unknown origin (29).

Evidence gaps persist because most studies have only focused on tobacco smoking-related COPD, leaving patients with other etiologies without a robust evidence-based characterisation in terms of clinical behavior, causes, molecular mechanisms, and prognosis (30).

## Risk factors

Several risk factors for COPD in never-smokers have been suggested (Table 1).

**Table 1:** Risk factors for COPD in never-smokers.

Category	Risk factors	References
Early-life and developmental factors	Low maximally attained lung function due to prenatal, childhood, or adolescent influences	(8, 26, 31–34)
Air pollution, occupational exposures and lifestyle factors	Indoor/outdoor air pollution for example: Smoke from biomass fuel (household), particulate matter (nitrogen dioxide, coal, biological and mineral dusts), occupational exposure to gases, fumes, or dust, (livestock farming, agriculture, sculptors, gardeners, warehouse workers) environmental tobacco smoke (passive smoking), dietary factors	(8, 16, 26, 35–37)
Infections	Tuberculosis, HIV infection, recurrent childhood respiratory infections	(8, 34, 38–40)
Asthma	History of asthma	(3, 4, 10, 14, 19, 34, 35, 38, 41–43)
Socioeconomic factors	Low socioeconomic status, associated with multiple risk factors for impaired lung growth and COPD	(8, 26)
Demographic and biological factors	Older age, genetic factors	(9, 10, 44, 45)

COPD: chronic obstructive pulmonary disease.

Categories are not mutually exclusive; several factors interact across the life course.

Early-life events such as premature birth and low-birth weight have become increasingly recognised risk factors (32–34). Childhood respiratory infections (e.g. pneumonia or bronchiolitis) along with exposure to air pollution have shown to impair normal lung growth in children. Suboptimal lung development early in life may predispose individuals to reduced lung function trajectories and thereby contribute to the development of COPD in adulthood (34, 46). Preterm birth may impair postnatal lung growth as this disrupts the normal physiological development of the respiratory system in utero. Maternal exposure to environmental pollutants, including tobacco smoke, can further affect the structural development of the postnatal airways (36). Taken together, tobacco exposure both in utero and during early life, increases the likelihood of developing an obstructive respiratory phenotype and consequently, the risk of COPD in later life (36). Smoking during pregnancy poses significant risks to the foetus by disrupting normal lung growth and development in utero. In addition, smoking-induced epigenetic modifications may prime the developing immune system, thereby increasing the risk of developing COPD in adulthood (34, 37).

Among never-smokers, air pollution represents the most widely recognised risk factor for COPD (8). Other reported contributors include exposure to environmental tobacco smoke, and various occupational inhalational exposures (8, 16, 35). Aside from active tobacco smoking, the main environmental exposures leading to COPD are, the inhalation of toxic particles and gases arising from both household and outdoor air pollution (14). Recent estimates suggest that up to 50% of the total attributable risk for COPD may be associated with air pollution exposure, a burden that is particularly pronounced in developing countries (8).

Respiratory infections are important contributors to the development of COPD. Tuberculosis (TB) in particular, is now recognised as an important and independent risk factor for chronic airflow obstruction (39). According to GOLD guidelines one of the newly presented etiotype is COPD-I (due to infections, including TB-associated COPD). This classification reflects the growing recognition that post-infectious structural and functional lung abnormalities constitute a distinct pathway to CAL (14).A family history for COPD is not only an independent risk factor for COPD development but also for frequent exacerbations and more severe disease (44). Expiratory airflow measurements, such as FEV_1_, FVC and the ratio FEV_1_/FVC show a hereditable pattern in COPD, which are related to specific genes (44). Genetic variants associated with reduced lung function and increased risk of COPD have also been identified. A clinically relevant, well known genetic risk factor for COPD is mutations in the SERPINA1 gene, which leads to hereditary deficiency of α-1 antitrypsin and increased risk for emphysema. This mutation affects only a small proportion of the global population (45, 47).

Multiple studies have shown that, among individuals who have never smoked, advancing age, and among women lower educational attainment are each independently associated with an increased likelihood of developing COPD (3). In addition, low body mass index (BMI) and increasing age have been identified as contributors to COPD risk (3, 16, 30, 35, 38).

Asthma is also known as a potential risk factor for the development of COPD (3, 4, 8, 29, 30, 35, 38, 41). A strong association between asthma and COPD has been shown in a Swedish birth cohort study (BAMSE) where a history of physician-diagnosed asthma was significantly related to the development of COPD (42). An Australian study showed that children with severe asthma had 32 times higher risk of developing COPD compared to controls (43). In a meta-analysis including 30 studies, children with asthma had an increased likelihood of developing COPD compared with those without asthma (34). In adults diagnosed with asthma a longitudinal study showed a 12-fold higher risk of developing COPD compared to those without asthma, after adjusting for smoking (14). Having both pneumonia and asthma during childhood further increases the risk of developing COPD in adulthood (40).

Lung function increases fast from birth until age 13–16 years, followed by a slower increase until a peak at age 20–25 years (Figure 1). Thereafter, lung function declines with normal ageing, without a distinct plateau phase in the mid-twenties. Consequently, the FEV_1_/FVC ratio decreases throughout life at a varying rate depending on age and sex (48). A person’s maximal attained lung function (FEV_1_ peak values) is a critical determinant of long-term respiratory health. The inability to reach normal peak FEV_1_ in early adulthood markedly increases the risk of an earlier onset of symptomatic airflow limitation and subsequent development of COPD, later in life (31, 49).

**Figure 1:**
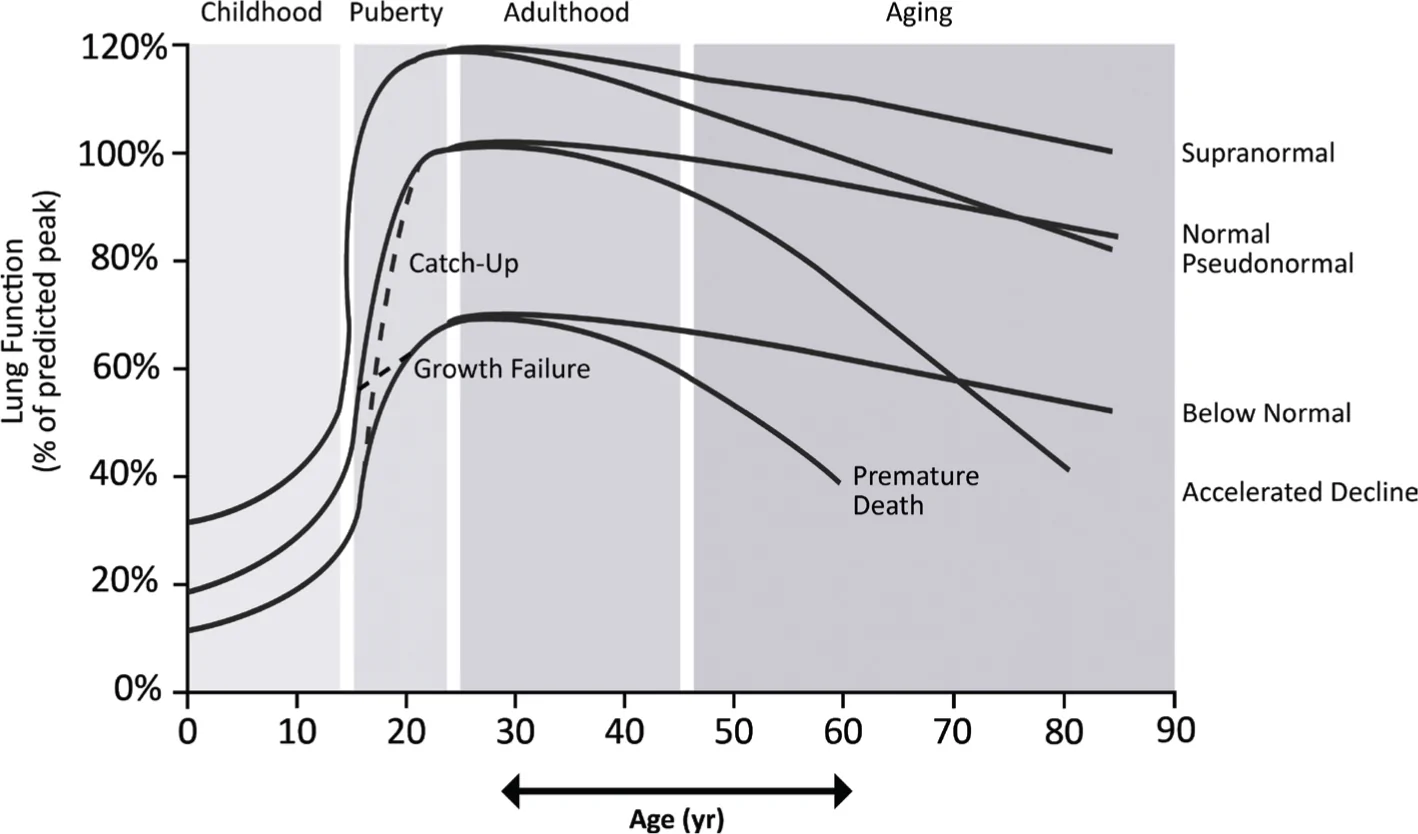
Trajectories of lung function, described as forced expiratory volume in 1 second of predicted value, (FEV_1_ %) over the life course. *(Modified from Agustí A, Hogg JC, N Engl J Med 2019, in GOLD Report 2026(14))*.

Preserved ratio impaired spirometry (PRISm) represents a spirometric pattern of prognostic value. It is defined by a normal FEV_1_/FVC ratio and a reduced FEV_1_ <80% of predicted value. PRISm is considered to be a subtype of lung growth impairment and is associated with an increased risk of COPD (50). Evidence from a recent Swedish longitudinal study showed that PRISm is related to development of airway obstruction regardless of smoking history (51).

In summary, COPD is understood to be a result from gene (G) environment (E) interactions occurring across the individual’s lifetime (T), a framework termed GETomics, including factors that can damage or disrupt the lungs and/or alter their normal development/ageing processes (14).

## Clinical characteristics

Several important differences exist between tobacco-related COPD and COPD that occur in never-smokers (25, 52). In Table 2, clinical characteristics of the two groups are presented.

**Table 2:** Clinical characteristics of tobacco-related COPD compared with COPD in never-smokers.

Clinical characteristics	Tobacco-related COPD	COPD in never-smokers
Young age of onset	+	++
Sex	Males > Females	Males < Females*
Airflow obstruction	+++	+
Hyperinflation	+++	+
Small airways obstruction	+	+++
Airway resistance	+	++
Sputum production	++	+
Dyspnoea	+++	+
Cough	+++	++
Reduction in DLCO	+++	0
Imaging: Emphysema	++	0
Imaging: air trapping	+	++
History of Asthma	+	++
Allergies**	+	++
FeNO levels, airborne allergens, eosinophil inflammation	+	++
FEV_1_ annual decline	++	+
Comorbidities	++	+
Exacerbations	+++	++
Effect of bronchodilators and inhaled steroids	++	Unknown
Impact on HRQL***	+++	+
History of tuberculosis	+	++

COPD: chronic obstructive pulmonary disease; DLCO: diffusing capacity of the lung for carbon monoxide FeNO: Fractional exhaled Nitric Oxide; FEV_1_: Forced expiratory volume in first second; HRQL: Health-related quality of life.

The plus signs illustrate an estimation of ordinal assessment of the variable’s presence, with more plus signs (+++) indicating a greater extent or higher level of that variable.

* In low- and middle-income countries: Females < Males

** Pollen and pets

*** COPD Assessment test, S:t George’s Respiratory Questionnaire

In never-smokers with COPD, the age of onset tends to be younger (25), airway obstruction is often less pronounced (21, 27) while small airways disease is more common (22), especially in those with a history of asthma (41). The sex distribution is relatively even with several studies reporting a female predominance in never-smokers (16, 19, 22, 25). In low- and middle-income countries, however, male predominance is more common. Never-smokers often report less sputum production, less cough (16, 25), and less exertional breathlessness (23). They also show normal diffusing capacity of the lung for carbon monoxide (DLCO) (25), and less emphysema (22, 25) as well as a slower decline in lung function over time (22). A recent Swedish study showed that never-smokers with COPD reported more respiratory comorbidities such as asthma and allergies, suggesting a type-2 inflammatory response (21, 23). Differences in COPD severity were shown in the burden of obstructive lung disease (BOLD) study. The proportion of patients with severe and very severe (GOLD stages III- IV) airway obstruction was significantly lower in never-smokers (6% vs. 14%) (3).

Co-occurrence of emphysema, CAL, and impaired DLCO is observed more frequently in current smokers than in never-smokers. Among never-smokers, emphysema is notably less prevalent compared with CAL and impaired DLCO, and only a very small proportion of never-smokers exhibit all three findings simultaneously (27) (Figure 2).

**Figure 2:**
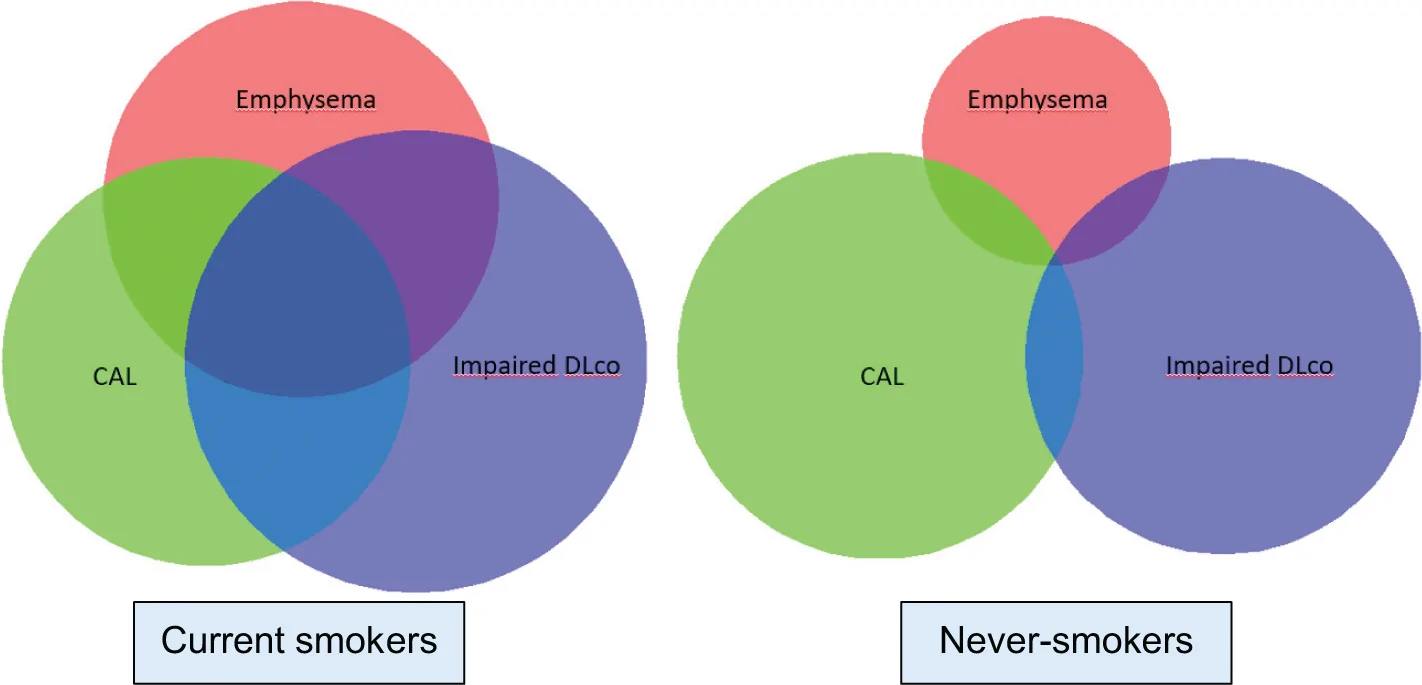
Venn diagram illustrating the distribution and overlap of emphysema, chronic airflow limitation (CAL), and impairment in diffusing capacity of the lung for carbon monoxide (DLCO) in current smokers and never-smokers. *(modified after Blomberg A, et al 2024 (27). The circles are proportional to the prevalences within the two groups*.

## Summary

COPD in individuals who have never smoked is common and increasingly recognised. Despite its growing recognition, research on COPD in never-smokers remains limited. Recent population-based data suggest that approximately one-third of all COPD cases worldwide occur in never-smokers, and emerging evidence indicates that these individuals represent a distinct clinical phenotype. Current understanding of the early-life factors, the environmental exposures, or respiratory illness that may predispose individuals to COPD remains incomplete. Clinically, never-smokers with COPD differ from those with smoking-associated disease in terms of symptom burden, degree of airway obstruction, comorbidities, and biomarker profiles.

Systematic reviews consistently highlight a substantial knowledge gap, particularly with respect to the natural history of the disease and optimal treatment strategies for this population. This lack of evidence is further compounded by the fact that never-smokers are consequently excluded from clinical trials, making it difficult to determine whether therapeutic recommendations derived from studies of smokers are applicable. Consequently, there is significant uncertainty regarding how best to tailor management for this phenotype. At present, never-smokers with COPD are treated according to the same guidelines as other patients with COPD, and when asthma is suspected or confirmed, treatment follows established asthma management principles.

Future research is needed to improve diagnostic evaluation in never-smokers with COPD and to develop personalised treatment strategies that better address their specific symptom profiles and underlying pathophysiology.
